# The Abortive Treatment of Acute Suppurative Inguinal Adenitis by Pressure-Bandage*Read before the Georgia Medical Association, Macon, April 21st.

**Published:** 1897-06

**Authors:** Henry R. Slack

**Affiliations:** LaGrange, Ga., Professor of Chemistry and Physics and Lecturer on Physiology in the Southern Female College


### THE ABORTIVE TREATMENT OF ACUTE SUPPURATIVE INGUINAL ADENITIS BY PRESSURE-BANDAGE.*

BY HENRY R. SLACK, PH.M., M.D., LAGRANGE, GA.,

Professor of Chemistry and Physics and Lecturer on Physiology in the Southern Female College.

There are few simple complications of chancroid and urethritis that worry the physician and patient more than an acute suppurating bubo. The classic treatment with tincture iodine, mercury, iodide ointment, belladonna ointment, etc., has, in my experience, been almost barren of results in aborting this trouble.

The practice of Auspitz of puncturing the inflamed gland before suppuration has taken place, and then introducing a probe and breaking up the glandular septa, so that the substance of the gland is discharged through the external wound, has found but few followers. It is, however, highly indorsed by Bumstead.

Taylor recommends the injection into their substance twenty minims of a solution of carbolic acid (8 gr. to the oz.) 0.5 gm. to 30 c. c. of water. This he claims invariably aborted suppuration.

Lydston advocates the early and complete extirpation of all buboes.

---

* Read before the Georgia Medical Association, Macon, April 21st.

The danger of infecting the glands is great by any of these methods, and, besides, they necessitate, for their successful practice, rest in bed. This is a requirement most seriously objected to by a large majority of patients in private practice. They want to be cured without being put to bed or the use of iodoform.

I must confess I had almost despaired of finding a safe and successful abortive treatment for bubo, until last winter, at the Johns Hopkins Hospital, in the genito-urinary clinic, I saw Dr. Gaither apply the pressure bandage.

I have seen it used in ten cases, most of which were in Dr. Gaither's clinic, though some were in my private practice, with the following results:

One patient complained that it increased the pain, which was so intense he could not bear for the bandage to remain on over a few hours. In another case, where fluctuation was distinctly felt, the suppuration continued and after the third day an incision was made. In the remaining eight the bubo was aborted, including two in which suppuration had begun. The following cases are typical:

CASE 1. C. B., white, age 18. November 16, 1895. Has had discharge from urethra three weeks. First attack. Some pain on micturation and increase in frequency. Discharge profuse and purulent. Has inflammatory bubo on left side, size of a horse-chestnut. Skin covering swelling red. Considerable pain on pressure, but no suppuration. Treatment: Injections bichloride mercury 1 to 20,000 and pressure bandage.

November 21. The swelling of the gland almost gone. No evidence of suppuration. Bandage reapplied.

CASE 2. A. P., colored, age 24. December 27, 1895. Patient has had bubo on left side for three weeks. He says it was preceded by two small discharging sores on either side of the frenum, which can still be seen, though nearly healed. There is a large inflammatory bubo in the left groin; glands are matted together and show evidence of suppuration over a small area. Skin covering swelling is adherent and glistening. Treatment: Pressure bandage applied.

January 2, 1896. Bubo smaller, harder and less painful. Signs of suppuration have disappeared. Bandage reapplied.

January 7. Bubo about gone, not painful. Bandage removed.

CASE 3. J. S., colored, age 25. Cook in hotel. October 6, 1896. Has slight discharge from urethra; inflammatory bubo on both sides. Right as large as hen's egg and so painful can hardly walk. Left about size of hickory-nut and painful on pressure, but no fluctuation in either. Skin covering glands adherent and glistening. Treatment: Balsam mixture and pressure bandage.

Patient said the bandage relieved the pain so much that he could walk with greater ease.

October 9. Complains that pain is more intense in right groin. The gland is larger and suppuration has begun. Left side, bubo is smaller, harder and all pain has disappeared. Reapplied pressure bandage.

October 20. Feels all right now. Right bubo smaller than a hickory-nut and left gland only slightly enlarged. No pain in either. Bandage removed.

Thus it will be seen that the bandage was a success in aborting the bubo in 80 per cent. of the cases in which used. The cases cited represent two gonorrheal and one chancroidal bubo, which was about the proportion treated.

Of course all buboes do not reach the suppurative stage. Whether treated or not, a good many will subside before suppuration begins.

Now, a word as to the method of applying this bandage, about which the text-books are very reticent. This cannot better be given than in Dr. Gaither's own language: "A piece of cotton as large as the fist is folded in on itself again and again, until it has the shape of the bubo, and when placed on it does not completely cover it. This is carefully adjusted and a wad of tightly compressed cotton as large as a cocoanut placed over it. Small pieces of cotton are also used on the inner and outer surfaces of the thigh to prevent chafing, and a very tight spica bandage put on."

The pressure bandage treatment for acute suppurative inguinal adenitis has, in my experience, been a succcss. It has the following points to recommend it above any other abortive treatment thus far suggested:

1. It is safe.
2. It generally reduces the pain.
3. It allows the patient to continue his usual avocation without interruption.
4. It gives a large number of successful cases.

